# Hematological Parameters from the Feline Blood Donor to the Blood Unit: What Changes Are to Be Expected?

**DOI:** 10.3390/ani12141819

**Published:** 2022-07-16

**Authors:** Marta Vascellari, Antonio Carminato, Giovanni De Zottis, Matilde Bisconti, Laura Gagliazzo, Elisa Bozzato, Valentina Bertazzo, Annalisa Stefani

**Affiliations:** 1 Canine and Feline Blood Bank Unit, Istituto Zooprofilattico Sperimentale delle Venezie, 35020 Legnaro, Italy; mvascellari@izsvenezie.it (M.V.); acarminato@izsvenezie.it (A.C.); gdezottis@izsvenezie.it (G.D.Z.); ebozzato@izsvenezie.it (E.B.); 2 Laboratory Medicine, Istituto Zooprofilattico Sperimentale delle Venezie, 35020 Legnaro, Italy; mbisconti@izsvenezie.it (M.B.); vbertazzo@izsvenezie.it (V.B.); 3 Statistical and Epidemiological Unit, Istituto Zooprofilattico Sperimentale delle Venezie, 35020 Legnaro, Italy; lgagliazzo@izsvenezie.it

**Keywords:** feline blood transfusion, sedation, hematological change, cat

## Abstract

**Simple Summary:**

Feline blood donation requires sedation to allow for good blood collection, avoiding venous damage and unnecessary donor stress. In the present study, we describe the variation of hematological parameters in a population of healthy blood-donor cats that underwent the same sedation protocol, including medetomidine, alfaxalone, and butorphanol. Significant differences in hematological parameters were observed between unsedated and sedated cats; particularly, the mean red blood cell count, hemoglobin concentration, hematocrit value, mean corpuscular volume, and red blood cells distribution width were significantly lower in sedated cats than in conscious ones, thus confirming that sedation is a critical procedure in cats. No significant differences for the main hematological parameters were observed between sedated cats and feline blood units, suggesting that the dilution with the conservative–anticoagulant solution (ratio 1:7) exerts negligible effects on these parameters with respect to samples of sedated animals.

**Abstract:**

Feline blood donation requires sedation to allow for good blood collection, avoiding venous damage and unnecessary donor stress. In the present study, we describe the variation of hematological parameters in a population of 74 healthy blood-donor cats that underwent the same sedation protocol, including medetomidine, alfaxalone, and butorphanol. Changes in hematological parameters were evaluated in blood samples collected from conscious cats (group A), sedated cats (group B), and feline whole-blood units (FBUs) (group C). Significant differences were observed between unsedated and sedated cats: the mean RBC count, HGB, HCT, and RDW were significantly lower in sedated cats than in conscious ones, with a difference of 17.95%, 18.42%, 28.21%, and 10.00%, respectively. In accordance with previously reported data, our results confirm that sedation is a critical procedure in cats that can affect most of the hematological parameters. The second finding of our study is that no significant differences for the main hematological parameters were observed between sedated cats and FBUs, thus suggesting that the dilution with the conservative–anticoagulant solution exert negligible effects on these parameters with respect to samples of sedated animals. This hematological change must be taken into consideration since such parameters are important to define the quality of FBUs.

## 1. Introduction

Feline transfusion medicine is a growing area of interest in veterinary medicine, due to the increasing availability of veterinary blood banks, which appropriately select feline blood donors and provide commercial feline blood units (FBUs), making transfusion therapy widely available to veterinarians. A conventional FBU contains a total volume of approximately 40–60 mL, including the anticoagulant conservative solution, with a volume ratio anticoagulant:blood of 1:7 [1].

In general, feline donors should be healthy, aged between 1 and 8 years, weigh at least 4.5 kg, and living indoors [1]. A complete history and physical examination, as well as hematology and serum biochemistry profiles, are recommended at screening, as well as at donation. Moreover, FeLV and FIV, hemotropic mycoplasma, and other vector-borne pathogens in endemic areas should be excluded by antigenic and molecular investigations [1]. Ideally, donors should be calm in temperament and easy to handle to reduce the level of sedation. In fact, sedation or general anesthesia is routinely used in cats to avoid unnecessary stress and to facilitate clinical procedures, including blood donation [1,2,3,4].

Several studies have reported the feasibility of different drug combinations and the effects induced by anesthetics on clinical parameters, particularly on the cardio-respiratory system [3,5,6]; in this regard, several studies have investigated the potential clinical and hematological alterations induced by sedation protocols in healthy cats, even though contradicting conclusions have sometimes been reported [6,7,8,9,10,11,12].

The quality of the FBU may be influenced by many factors, starting with the selection of donors and the method of blood collection. Particularly, red blood cells (RBCs) and hemoglobin (HGB) content in the FBU are critical parameters that may influence the efficacy of the transfusion in the recipient. However, while the physical criteria for selection of feline blood donors are established, no threshold values for hematological parameters of the blood donor, as well as for the FBU, have been defined to date.

Few studies conducted in blood bank settings have investigated the hematological values in FBU soon after blood collection or during the storage period [13,14,15]. To the best of our knowledge, no previous studies investigated changes of hematological parameters occurring in blood donors before and after sedation, as well as in FBU.

The aim of this study was to investigate the effect of sedation on hematological parameters of selected feline blood donors, as well as the effect of the dilution with the conservative anticoagulant solution in the final FBU.

## 2. Materials and Methods

### 2.1. Animal Selection and Sampling Protocol

The study population consisted of healthy owned cats that weighed at least 4.5 kg and were included in the blood donors register of the feline blood bank of the Istituto Zooprofilattico Sperimentale delle Venezie (IZSVe), Legnaro (Italy).

All cats were clinically healthy and tested negative for feline immunodeficiency virus (FIV), feline leukemia virus (FeLV), *Mycoplasma* spp., *Babesia* spp., *Cytauxzoon* spp., and enteric parasites via antigenic and molecular assays. The animals were regularly vaccinated and treated against endo- and ectoparasites.

Written owner consent was required to authorize sedation, blood collection, and the exploitation of data for scientific purposes.

Blood samples were collected from conscious cats, sedated cats, and FBU, as described below:

Group A: 2 mL of blood was collected from 65 conscious cats through jugular venipuncture by a 21G needle and immediately transferred into K3-EDTA tubes.

Group B: 2 mL of blood was collected from 40 sedated cats through cephalic or femoral venipuncture by a 21G needle and immediately transferred into K3-EDTA tubes.

Group C: 50 mL of blood was collected from 21 sedated cats, from the jugular vein, using a butterfly 21G, collected to the TEC724 closed blood collection kit for cats (Futurlab, Italia) [14] and mixed with 8 mL of citrate–phosphate–dextrose–adenine (CPDA-1) anticoagulant. After gentle mixing, 2 mL of blood was sampled through a self-cleaning blood bag valve, using a sterile plain vacutainer tube.

Cats from both groups B and C underwent the same sedation protocol, which consisted of an intramuscular injection of medetomidine (Domitor; Pfizer, New York, NY, USA) 5–10 mcg/kg, alfaxalone (Alfaxan; Vétoquinol, Lure, France) 0.5–0.7 mg/kg, and butorphanol (Morphasol; Graeub, Bern, Switzerland) 0.1–0.24 mg/kg, mixed together in the same syringe.

### 2.2. Hematological Analysis

All samples were maintained at a refrigeration temperature and within 12 h from sampling.

The hematological analysis was performed on K3-EDTA and CPDA-1 samples, using a XN-1000 Vet analyzer (Sysmex Europe SE, Norderstedt, Germany).

The evaluated hematological parameters included red blood cell count (RBC, M/µL); hemoglobin concentration (HGB, g/dL); hematocrit (HCT, %); mean corpuscular volume (MCV, fL); mean corpuscular hemoglobin (MCH, pg); mean corpuscular hemoglobin concentration (MCHC, g/dL); red blood cell distribution width (RDW, %); platelet count (PLT, K/µL); and total white blood cell count (WBC, K/µL).

Intermediate and total precision were calculated with a pool of two randomly selected feline specimens (N = 20) and a manufacturer’s quality control material (N = 2 × 5), respectively, and are described by a coefficient of variation (CV) less than 5% for all evaluated parameters. 

The platelet count was excluded from statistical analysis because of the presence of clumps in several samples, as detected in blood smears routinely prepared and stained with a Wright/Giemsa automatic stainer (Aerospray, Delcon, Italy).

The plasma H index was used to determine the free HGB concentration in mg/dL, using a clinical chemistry analyzer (Cobas C501, Roche Diagnostics GmbH, Mannheim, Germany) in order to calculate the percentage of hemolysis, as previously described [14].

### 2.3. Statistical Analysis

To explore data distribution and to identify possible outliers, a descriptive analysis was performed. The blood sample was considered the statistical unit.

The effects of the Groups (A, B, and C), sex (female and male), two age classes (≤48 and >48 months), and their interaction on hematological parameters distribution were assessed by using a general linear model (GLM). The residual diagnostics were used to evaluate the model’s goodness of fit.

For MCHC, given non-normal data distribution, a non-parametric test (Kruskal–Wallis test) was performed.

Statistical significance was set to *p* < 0.05. The statistical analyses were performed by using StataBE 17 (Stata Corp. LP, College Station, TX, USA).

## 3. Results

Overall, 172 blood samples obtained from 74 cats (females = 26; male = 48) were included in the study. All cats were of blood type A. Fifty-five cats were mixed breed, while nineteen were purebreds. The age ranged from 7 to 106 months and was 53 months on average. Detailed characteristics of the feline population (sex, age, and breed) and hematological data are reported in Supplementary Table S1.

Seventy-eight blood samples belonged to group A (N = 65 conscious cats), 65 to group B (N = 40 sedated cats), and 29 to group C (N = 21 FBU). 

The GLM showed that gender and age had no significant effects on the hematological parameters.

For each group, the mean values and standard deviation (SD) of RBC (M/µL), HGB (g/dL), HCT (%), MCV (fL), MCH (pg), MCHC (g/dL), RDW (%), and WBC (K/µL) are summarized in Table 1. The calculated percentage of hemolysis [14] was negligible in all samples, always below the most stringent regulatory guidelines, fixed at <0.8% by the Council of Europe Guide for human blood components [16].

Significant differences in RBC (*p* < 0.001), HCT (*p* < 0.001), MCV (*p* < 0.001), MCHC (*p* < 0.001), RDW (*p* < 0.001), and WBC (*p* = 0.001) were observed among the three groups.

Particularly, the mean RBC count, HGB concentration, HCT, and RDW were significantly lower in group B than in group A, with a difference of 17.95%, 18.42%, 28.21%, and 10.00%, respectively (*p* < 0.001). Similarly, the same parameters were significantly lower in group C than in group A, with a difference of 26.03%, 26.17%, 29.84%, and 16.47%, respectively (*p* < 0.001). On the contrary, no significant differences were observed between group B and C for the abovementioned parameters. 

The mean MCV was significantly lower in group B (41.1 ± 3.9 fL) than in group A (44.9 ± 4.9 fL), showing a difference of 8.74% (*p* < 0.001), while no significant changes were observed between group A and C, as well as between group B and C.

Significantly higher values of MCHC were registered in group B (35.9 ± 3.0 g/dL) than in group A (33.0 ± 3.0 g/dL), with a difference of 7.56% (*p* < 0.001), as well as in group B compared to group C (34.0 ± 1.0 g/dL), with a difference of 5.31% (*p* < 0.001). No significant differences were observed between groups A and C.

Finally, the mean WBC count was significantly lower in group B (7.21 ± 2.73 K/µL) compared to group A (9.01 ± 3.28 K/µL), showing a difference of 27.40% (*p* = 0.005), as well as between group C (6.86 ± 2.93 K/µL) and group A, with a difference of 36.76%.

## 4. Discussion

Feline blood donation is a short-term and minimally invasive procedure. Notwithstanding, sedation must be applied to allow good collection of blood, avoiding venous damage and unnecessary donor stress. Ideally, a desirable sedation protocol should provide good quality of sedation, quick recovery, and minimal side effects on the cardiovascular system, as well as on hematological variables [5]. 

Different combinations of drugs are considered feasible for sedation in feline blood donors [1,5], and the choice of the sedation protocol should take into consideration specific advantages and disadvantages. Particularly, drugs causing significant cardiorespiratory depression and hypotension should be avoided [1].

In the present study, we described the variation of hematological parameters in a population of healthy blood-donor cats that underwent the same sedation protocol, including medetomidine, alfaxalone, and butorphanol. In our survey, all the cats showed good sedation, allowing easy blood collection and quick recovery. No cats experienced adverse effects and/or severe cardiovascular depression hindering the withdrawal. 

On the other hand, significant variations in some hematological parameters were registered between conscious and sedated cats. Particularly, the mean RBC count, HGB, HCT, MCV, and RDW were significantly lower in sedated cats than in conscious ones. 

Several previous studies have reported the effects of different sedation protocols on hematological parameters in cats [5,6,7,8,9], sometimes with contradicting results. 

Biermann et al. [5] investigated the effect of four different protocols, namely midazolam and butorphanol (MB); midazolam, butorphanol, and ketamine (MBK); midazolam, butorphanol, and dexmedetomidine (MBD); and ketamine and dexmedetomidine (KD), on six healthy cats. When compared to the baseline, MBK, MBD, and KD caused significant hematological changes, with decreased RBC counts, PCV, HGB concentration, and MCV [5]. Similarly, in one more study, pre- and post-induction hematological parameters were evaluated in 12 cats, using intravenous ketamine and midazolam, and intramuscular buprenorphine [6]. On average, the RBC count, HGB concentration, and HCT were significantly decreased in post-induction samples, being 24.7%, 23.8%, and 24.9% lower than in pre-induction samples, respectively [6].

One further study showed a significant decrease in packed cell volume (PCV) and hemoglobin in a group of healthy cats, following intramuscular pre-anesthetic sedation with three different protocols, namely methadone–acepromazine, methadone–dexmedetomidine, or methadone–midazolam–alfaxalone [7].

Even if the sedation protocols applied in our study and in the previous ones are different, the obtained results are similar, thus highlighting a significant effect of sedative drugs on hematological parameters soon after inoculation. There are several mechanisms to explain these findings, including a decrease in vascular resistance resulting in vasodilation, and extravascular pooling of red cells due to alterations in hemodynamic function. The main substantiate hypothesis is the sequestration of erythrocytes in splenic sinuses due to the relaxation of smooth muscles, leading to a decrease of circulating erythrocytes in the peripheral blood [6].

On the other hand, it should be considered that the acute stress caused by the handling of conscious cats for blood collection could have increased the circulatory pool of erythrocytes and leukocytes in this group. In fact, it is known that catecholamine, such as epinephrine and norepinephrine, associated with excitement or fear, are responsible for splenic contraction in many species, and can alter the distribution of leukocytes, particularly in cats [6].

Different from the above reported results, the hematologic and hemostatic parameters evaluated in 50 cats subjected to physical restraint and then randomly divided into two groups of 25 animals, receiving dexmedetomidine and butorphanol (DB group) or dexmedetomidine, butorphanol, and ketamine (DBK group), showed no statistically significant differences between pre- and post-sedation groups [8]. The authors concluded that both protocols were effective for short-duration chemical restraint, causing no clinically relevant effects [8]. Similarly, the effects of intravenous the low-dose ketamine-diazepam combination that was used for short-duration chemical restraint were evaluated in 42 client-owned cats. Even if significant changes were observed for most of the analytes tested (complete blood count, biochemical profile, and coagulative profile), just prior to and just after sedation, the authors concluded that the magnitude of the observed changes was not of clinical relevance [9].

Previously reported data recorded inconstant results on the effect of sedation on MCV values [7,8,10,11]; in our survey, the mean MCV significantly decreases after sedation, showing lower values in sedated than in conscious cats. This finding suggests that sedation could induce an increase in resistance of peripheral small vessels, resulting in a redistribution of the erythroid compartment. When MCV was measured in FBU, the mean value was not different from those of not-sedated cats, probably due to the osmotic effect of the CPDA-1 solution. However, no reliable scientific hypotheses have been formulated so far to explain this phenomenon.

Both MCH [HGB(g/dL)/RBC(10^6^/μL)] and MCHC [HGB(g/dL) × 100/HCT(%)] are calculated values. HCT is a calculated result itself [MCV(fL) × RBC(10^6^/μL)/10] that takes into account the volume of RBC. Thus, the changes in MCH and MCHC are dependent on changes of RBC, HGB, and MCV.

The second finding of our study is that no significant differences for the main hematological parameters were observed between sedated cats and FBU, thus suggesting that the dilution with the conservative–anticoagulant solution (ratio 1:7) exerts negligible effects on these parameters with respect to the samples of sedated animals. To the best of our knowledge, only one previous study investigated hematological changes between feline blood donors and FBU, showing a significant decrease in RBC count, HGB concentration, HCT, RDW, and WBC between seven blood donors sedated with a combination of tiletamine and zolazepam, and blood units [13]. The authors attributed these changes to the combined dilution effect due to the amount of the anticoagulant preservative solution (CPDA-1) and the endovenous fluid therapy applied during the blood donation [13]. 

In our opinion, in light of the results herein obtained, the 1:7 anticoagulant:blood ratio does not cause significant hematological changes and is appropriate to maintain a good quality of FBU during the entire storage period, as previously demonstrated [14]. 

A possible limit of our study is represented by different sites of sample collection between group B (femoral or cephalic vein) and C (jugular vein). Notwithstanding, while the effects of needle gauge on hemostatic indices have been investigated in cats [17], to the best our knowledge, no data are available in the literature about the possible effects of the vein district or caliber on the measured hematological parameters.

## 5. Conclusions

In conclusion, this study included a large series of samples, confirming that sedation in cats is a critical procedure which can affect most of the hematological parameters. HGB concentration, RBC count, and HCT were the parameters that were mostly affected, being significantly lower after sedation. Even though the vast majority of the results obtained was within the feline reference intervals, we observed that the RBC count and HGB concentration dropped below the minimal threshold level in several FBU. This occurrence must be taken into strong consideration, since such parameters are important to define the quality of a FBU, which may influence the efficacy of the transfusion in the recipient. Since any sedation protocol may have different effects on hematological parameters, further investigations should be devoted to establishing a standardize sedation protocol to ensure the safety of donation, as well as the quality of the donated FBU.

## Figures and Tables

**Table 1:** Hematological parameters (mean ± SD; minimum and maximum values) evaluated in healthy donor cats before (group A, N = 78) and after (group B, N = 65) sedation and in whole blood donated units (group C, N = 29).

Parameter (Laboratory Reference Interval)	Group	Mean	SD	Min–Max	
RBC (5.10–10.0 M/µL)	A	9.09 ^a^	1.32	5.16–12.05	***
	B	7.64 ^b^	1.33	4.79–10.07	
	C	7.42 ^b^	1.00	5.79–10.13	
HGB (8.00–15.00 g/dL)	A	13.35 ^a^	1.91	8.68–18.10	***
	B	11.42 ^b^	2.24	7.23–20.00	
	C	10.91 ^b^	1.85	8.10–17.20	
HCT (30.0–45.0 %)	A	40.6 ^a^	5.7	26.7–57.9	***
	B	31.4 ^b^	5.2	23.1–43.1	
	C	32.1 ^b^	5.4	24.3–49.7	
MCV (39.0–55.0 fL)	A	44.9 ^a^	4.9	35.1–57.7	***
	B	41.1 ^b^	3.9	32.9–49.6	
	C	43.3 ^a,b^	3.5	35.2–49.1	
MCH (13.0–17.0 pg)	A	14.7	1.3	12.0–17.6	ns
	B	14.7	1.1	11.4–17.5	
	C	14.7	1.2	12.5–17.0	
MCHC (30.0–36.0 g/dL)	A	33.0 ^b^	3.0	27.2–42.8	***
	B	35.9 ^a^	3.0	30.6–44.6	
	C	34.0 ^b^	1.0	32.0–35.8	
RDW (16.0–23.0 %)	A	19.7 ^a^	1.6	16.8–24.0	***
	B	18.1 ^b^	2.0	13.2–23.0	
	C	17.1 ^b^	1.5	13.6–19.7	
WBC (5.00–19.00 K/µL)	A	9.01 ^a^	3.28	3.97–18.49	**
	B	7.21 ^b^	2.73	2.12–14.63	
	C	6.86 ^b^	2.93	3.43–17.04	

RBC, red blood cell; HGB, hemoglobin; HCT, hematocrit; MCV, mean cell volume; MCH, mean corpuscular hemoglobin; MCHC, mean corpuscular hemoglobin concentration; RDW, red cell distribution width; WBC, with blood cell. ** Significant at *p* < 0.01, *** significant at *p* < 0.001, ns = not significant, and ^a,b^ values with different superscripts are significantly different between groups at least at *p* ≤ 0.005.

## Data Availability

The data presented in this study are available upon request from the corresponding author.

## Supplementary Materials

The following supporting information can be downloaded at https://www.mdpi.com/article/10.3390/ani12141819/s1. Table S1: Characteristics of the feline population (sex, age, and breed) and hematological data. Outlier data are highlighted in yellow.
